# Prognostic Impact of the Angiogenic Gene *POSTN* and Its Related Genes on Lung Adenocarcinoma

**DOI:** 10.3389/fonc.2022.699824

**Published:** 2022-06-27

**Authors:** Dongfeng Sun, Zhibo Gai, Jie Wu, Qingfa Chen

**Affiliations:** ^1^Department of Thoracic Surgery, The First Affiliated Hospital of Shandong First Medical University and Shandong Provincial Qianfoshan Hospital, Shandong Medicine and Health Key Laboratory of Emergency Medicine, Shandong Lung Cancer Institute, Shandong Institute of Respiratory Diseases, Jinan, China; ^2^Department of Clinical Pharmacology and Toxicology, University Hospital Zurich, University of Zurich, Zurich, Switzerland; ^3^Department of Pathology, The Affiliated Hospital of Qingdao University, Qingdao, China; ^4^Institute of Tissue Engineering and Regenerative Medicine, Liaocheng People’s Hospital, Liaocheng, China

**Keywords:** lung adenocarcinoma, prognosis, competing endogenous RNA, angiogenesis, angiogenesis-related genes

## Abstract

**Background:**

The function of angiogenesis-related genes (ARGs) in lung adenocarcinoma (LUAD) remains poorly documented. This study was designed to reveal ARGs in LUAD and related networks.

**Methods:**

We worked with sequencing data and clinical information pertaining to LUAD from public databases. ARGs were retrieved from the HALLMARK_ANGIOGENESIS gene set. Differential analysis and Kaplan–Meier (K–M) analysis were performed to authenticate the ARGs associated with LUAD. Weighted gene correlation network analysis was performed on the mining hub genes linked to the abovementioned genes, and functional enrichment analysis was done. Subsequently, Cox regression analyses were used to construct the prognostic gene. *POSTN* and microvessel density were detected using immunohistochemistry.

**Results:**

*POSTN*, an ARG that was highly expressed in patients with LUAD and was closely associated with their weak overall survival was identified. Differentially expressed genes associated with *POSTN* were mainly enriched in entries related to the tubulointerstitial system, immune response, and epithelial cells. A positive correlation was demonstrated between *POSTN* expression and tumor microvessel density in LUAD. Subsequently, a prognostic gene signature was constructed and revealed that 4 genes may predict the survival of LUAD patients. Furthermore, the ESTIMATE and CIBERSORT analyses suggested that our risk scoring system may be implicated in altering the immune microenvironment of patients with LUAD. Finally, a ceRNA network was constructed based on the prognostic genes, and the regulatory networks were examined.

**Conclusion:**

*POSTN*, a novel prognostic gene signature associated with ARGs, was constructed for the prognosis of patients with LUAD. This signature may alter the immune microenvironment by modulating the activation of the tubulointerstitial system, epithelial cells, and immune cells, ultimately affecting patient survival.

## Introduction

Lung cancer is the leading cause of cancer-related deaths worldwide (1). The incidence of lung adenocarcinoma (LUAD), which has a high recurrence, high metastasis potential, and poor prognosis, is increasing (2, 3). The diagnosis of early-stage lung cancer has now become a challenge for radiologists and oncologists (4, 5). Recently, tumor microvascular computed tomography imaging signs have become an important diagnostic basis in the early stage (adenocarcinoma *in situ* and minimally invasive adenocarcinoma) of LUAD (6), which indicates that angiogenesis is an early event in LUAD. As the tumor progresses, the tumor cells absorb more nutrients from the body, thus activating the signal pathways associated with angiogenesis and releasing angiogenesis-related factors (7). Finally, more abnormal blood vessels are formed in and around the tumor, which promotes tumor metastasis. In addition, tumor treatment is closely related to the formation of abnormal tumor blood vessels. The abnormal blood vessels and the damaged perfusion often restrict the cytotoxic drugs and the immune cells in the systemic circulation from entering the tumor tissues. Thus, the tumor can evade the immune system (8). Abnormal tumor vascularization also induces a series of changes such as tumor stem cell programming and epithelial–mesenchymal transition (9). Therefore, tumor angiogenesis is considered as a necessary condition for tumor growth and progression (10, 11) and has been identified as an important therapeutic target for many cancers (12). Hence, it is of great significance to decipher the relationship between angiogenesis and the associated gene changes in LUAD.

Tumor angiogenesis is chiefly mediated by the vascular endothelial growth factor–vascular endothelial growth factor receptor signaling pathway and the related tumor microenvironment (TME) (12). Anti-angiogenesis therapy is a vital part of cancer treatment. Anti-vascular therapy combined with other targeted therapies or immunotherapy can provide additional treatment options for patients with a malignant tumor. However, angiogenesis-related factors and regulatory pathways are very complex, and it is difficult to identify the key angiogenic genes in tumor development. This difficulty particularly applies to the angiogenesis-related genes involved in the entire process of tumor development and could be used as a diagnostic marker for an early-stage tumor or as a target for anti-vascular therapy in patients with an advanced tumor.

This study aimed at revealing the expression and the complex connection of angiogenesis-related genes in LUAD. We obtained the sequence data and clinical information of LUAD from large public databases and analyzed the genes involved in angiogenesis. We attempted to identify an angiogenesis-related gene that could be used as a diagnostic and therapeutic target for LUAD. We also intended to construct a novel prognostic gene marker associated with angiogenesis (Arg) which may alter the immune microenvironment and ultimately affect the survival of patients with LUAD. Our work is expected to lay the foundation for a comprehensive study on the mechanism of abnormal angiogenesis in LUAD.

## Materials and Methods

### Data Source

A total of 560 samples were obtained from The Cancer Genome Atlas (TCGA) database for second-generation sequencing data, including 58 normal samples and 502 tumor samples. Data on miRNA mature sequencing was also downloaded from the TCGA-LUAD database, which covered 45 normal samples and 513 tumor samples. In the TCGA database, 446 LUAD samples containing complete survival information were enrolled in the evaluation and validation analysis of the prognostic model. In addition, the GSE26939 dataset containing 116 LUAD samples acquired from the Gene Expression Omnibus database was considered as an external validation set. A total of 115 LUAD samples containing the complete survival information were included in the validation analysis of the prognostic model.

For 36 ARGs, they were obtained from the HALLMARK_ANGIOGENESIS gene set (**Supplementary Table S1**).

### Differential Analysis

The differential analysis in the TCGA database was implemented by executing the limma package in R. Genes meeting |log_2_ fold change (FC)| > 1, *P* <0.05, were considered as differentially expressed genes (DEGs).

### Screening for Differentially Expressed ARGs Associated With Survival in LUAD Patients

The intersection analysis was applied to ascertain the differentially expressed ARGs. Subsequently, a univariate Cox regression analysis was performed in the survival package, and differentially expressed ARGs (DE-ARGs) with *P* <0.05 were subjected to the K–M analysis. Similarly, genes with *P* <0.05 were assumed to be the key genes.

### Immunohistochemical Staining and Analysis of the *POSTN* Expression and MVD

A total of 64 patients who underwent lobectomy or sublobectomy and were diagnosed with LUAD in The First Affiliated Hospital of Shandong First Medical University were recruited into the study. The samples of tumor and adjacent normal tissues were acquired from these patients. This study was approved by the ethics committee of The First Affiliated Hospital of Shandong First Medical University and Shandong Provincial Qianfoshan Hospital (YXLL-KY-2020-081), and written informed consent was obtained from all patients. POSTN antibody (19899-1-AP; Proteintech Group, Wuhan, China) and CD34 (60180-2-Ig; Proteintech Group, Wuhan, China) were used in this study. According to the manufacturer’s instructions, immunohistochemical staining was performed for the section by using the Envision+ DAB kit (Dako, Glostrup, Denmark). These sections were incubated with phosphate-buffered saline instead of a primary antibody and served as a negative control. A semi-quantitative method was employed to measure the staining intensity of POSTN as described elsewhere (13). Briefly, 10 fields of view were observed at ×400 magnification, and 100 tumor or alveolar epithelial cells were counted per field of view. The positive cell ratio was scored as follows: 0 point (<5% positive cells), 1 point (5–25%), 2 point (25–50%), 3 points (50–75%), and 4 points (>75%). The staining intensity scores were as follows: 0 points (unstained), 1 point (light-yellow), 2 points (yellowish-brown), and 3 points (dark brown). The product of the positive cell score and the stain intensity score was the final score of staining. Although CD34 is not a specific marker for evaluating angiogenesis, it is the most reliable marker for endothelium. The number of microvessels in the tumor was measured by CD34 staining, defined as clear brown or dark brown particles on the cytoplasm and/or cell membrane, which may include stained microvessels, individual endothelial cells, or cell masses. If they have a distinct border with adjacent tumor cells and surrounding connective tissue, they are considered microvessels. The entire section is examined at low magnification, and three “hot spots” with high concentrations of positive-staining blood vessels were found. The number of microvessels was then calculated at high-power field-of-view. If the lumen diameter is larger than the red blood cell or a smooth muscle is observed in the vessel wall, this type of vessel is excluded. The mean number of microvessels in five visual fields was taken as the microvessel density (MVD) of the sample.

### WGCNA

The expression data profile of genes with FPKM >1 from TCGA was transformed into a gene co-expression network using a weighted gene correlation network analysis (WGCNA) package in R. We first evaluated the quality for the expression data matrix of the TCGA-LUAD database (**Supplementary Figure S1A**). To incorporate similar modules obtained by the dynamic cut-tree algorithm, we set MEDissThres to 0.6 (**Supplementary Figures S1B, C**). All genes were identified for the heat map (**Supplementary Figure S1D**). The expression of the key genes was cited as a phenotype to target the key modules. Next, two metrics were calculated for the genes in the black modules: one was the Pearson correlation coefficient for the genes in the module and the first principal component of the module, called the module membership (MM), and the other was the Pearson correlation coefficient of the genes in the sample with the phenotype, called the gene significance (GS). Overlapping genes between TCGA-DEGs and the key module genes were identified as key gene-related-hub DEGs.

### Functional Enrichment Analysis

All functional enrichment analyses were based on key gene-related-hub DEGs. This project was performed using the R software clusterProfiler (14) package (Version 3.18.0) for the Kyoto Encyclopedia of Genes and Genomes (KEGG) and Gene Ontology (GO) enrichment analyses. *P* <0.05 was considered to indicate statistical significance.

Moreover, the canonical pathway and disease and function enrichment analyses were also implemented using the Ingenuity Pathway Analysis (IPA) which could potentially uncover the hidden biological significance and the value of the data in terms of the biological pathways (15). Furthermore, the IPA was employed to unravel the interactions between the molecules.

### Construction and Assessment of Risk Models

The TCGA-LUAD database was randomly divided into a training set and a testing set (7:3 ratio). To screen for genes that were strongly associated with patient overall survival (OS), a K–M analysis was performed on the key gene-related-hub DEGs in the training set, where genes with *P* <0.05 were included in a univariate Cox regression analysis. Similarly, genes fulfilling these conditions were used for multivariate Cox regression analyses. The final output genes were used to construct a prognostic model. The risk scores were further calculated for each sample in the training, testing, and external validation sets based on the coefficients and expression of each prognostic gene. The patients in each dataset were categorized into high- and low-risk groups depending on their respective median risk score values. K–M analysis was applied to assess the differences in OS between patients in the high- and low-risk groups. A subsequent receiver operating characteristic (ROC) analysis was performed to test the prognostic validity of the risk model.

### Independent Prognostic Analysis

The identification of independent prognostic factors was based on univariate and multivariate Cox regression analyses. *P* <0.05 was considered to indicate statistical significance. In this study, age, gender, tumor stage, American Joint Committee on Cancer (AJCC) pathological stage, and AJCC pathological TNM stage were subjected to independent prognostic analysis along with the risk score as the patients’ clinical characteristics. Finally, a nomogram was constructed with variables showing *P* <0.05 from the results of the multivariate Cox analysis, and calibration curves were plotted to view the predictive prognostic capability at 1, 3, and 5 years. A decision curve analysis (DCA) was then presented to the clinical benefit of the nomogram. In addition, the C-index for the nomogram was set to 0.75.

### Evaluation of Tumor Microenvironment

An ESTIMATE algorithm was deployed to calculate the immune score and the stromal score as described elsewhere (16), which was used to project the total immune and stromal infiltrations. The ESTIMATE score represented a mixture of the two scores mentioned above and mirrored the purity of the tumor. To anticipate the proportion of immune-infiltrating cells, the expression matrix was uploaded to the CIBERSORT and calculated according to the LM22 signature with 1,000 permutations (17). The effective samples were picked at *P* <0.05 (18).

### Construction of ceRNA Network

The DE-lncRNA–DE-miRNA–mRNA ceRNA network was constructed based on the theory that lncRNA can regulate the mRNA activity by directly invoking the miRNA sponge (19). For the miRNAs in the network, which were obtained in the TCGA-LUAD database with the previously described differential analysis method, there were 428 DE-miRNAs, of which 251 were upregulated and 177 were downregulated genes (**Supplementary Figure S2**; **Supplementary Table S2**). In addition, 43 DE-lncRNAs identified from the previously obtained 2,028 DEGs are exhibited in **Supplementary Table S3**. The starBase database was used to forecast the binding relationships of the abovementioned DE-miRNAs and DE-lncRNAs. The negatively regulated relationship pairs were retained. The transcriptome sequences of the prognostic genes and DE-miRNAs were also obtained from the National Center for Biotechnology Information and miRbase, respectively. Next, the possible binding relationships between the prognostic genes and the DE-miRNAs screened above were predicted using the miranda (20) software (version 3.3a), and the binding score was set to 150. Similarly, prognostic gene–miRNAs pairs with negative regulatory relationships were revealed. The ceRNA network was sketched with Cytoscape v3.8.2.

### Statistical Analysis

The Venn diagram was plotted in the Jvenn (21) (http://jvenn.toulouse.inra.fr/app/example.html) webapp. In both univariate and multivariate Cox analyses, variables with a hazard ratio (HR) >1 were regarded as risk factors and, conversely, as protective factors. Chi-square and rank-sum tests were employed to analyze whether the clinical traits were associated with the high- and low-risk groups. Data analysis was achieved in R software if not stated otherwise, and *P* <0.05 was considered to indicate the threshold of significance.

## Results

### Identification of ARGs Related to LUAD

**Figure 1:**
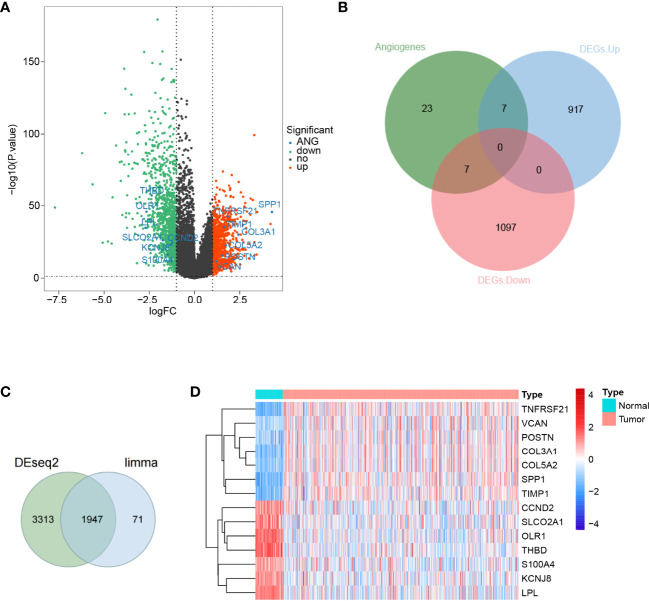
Differential expression of angiogenesis-related genes in lung adenocarcinoma. **(A)** A volcano map of gene differential expression in lung adenocarcinoma and normal lung tissue samples. The abscissa represents log_2_FC and the ordinate represents -log_10_(*P*-value). Each dot represents a gene, and the red dots represent upregulated differentially expressed genes. The green dots represent downregulated differentially expressed genes, while the black dots represent no significant differences. The horizontal reference line represents *p*-value = -log_10_(0.05), and the vertical reference line represents log_2_FC = ± 1. **(B)** Fourteen angiogenic-related genes were screened, of which 7 were upregulated and another 7 were downregulated. **(C)** Limma and Deseq2methods for gene calculations. In total, 96% of the differentially expressed genes calculated by Limma overlapped withDeseq2. **(D)** A total of 14 angiogenic differential gene expression heat maps.

To discriminate the DEGs between the LUAD and normal subjects in the TCGA database, we assumed the absolute value of log_2_FC >1 and *P*-value <0.05 as the threshold levels of significance. A total of 2,028 DEGs, including 924 upregulated genes and 1,104 downregulated genes, were appraised compared to normal tissues (**Supplementary Table S4**). The volcano plot of the DEGs is illustrated in **Figure 1A**. Subsequently, we obtained 36 ARGs from the HALLMARK_ANGIOGENESIS gene set. From this set of DEGs, a total of 14 DE-ARGs were extracted (**Figure 1B**), including seven significantly upregulated DE-ARGs and seven significantly downregulated DE-ARGs. Two different ways for gene calculations (by Limma and Deseq2) are shown in **Figure 1C**. Deseq2 yields 5,360 genes, and Limma yields 2,028 genes. In total, 96% of the differentially expressed genes calculated by Limma overlapped with Deseq2. A heat map portraying the expression levels of these genes is shown in **Figure 1D**.

### POSTN Is a Pivotal Gene in the Prognosis of Patients with LUAD

**Figure 2:**
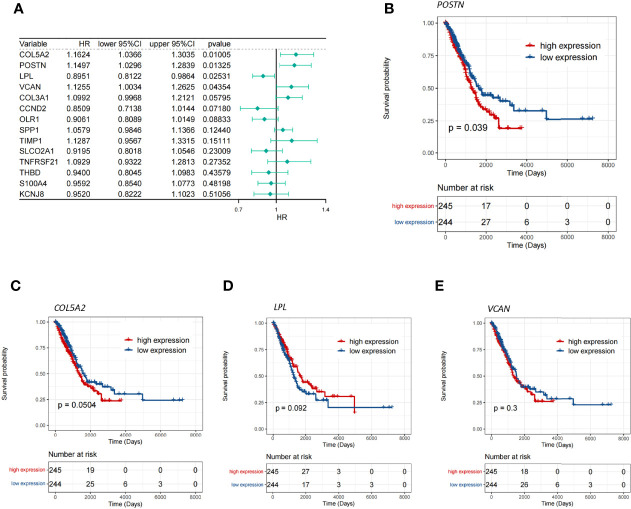
Screening of the key genes for angiogenesis. **(A)** Single-factor Cox analysis of angiogenic differentially expressed genes. HR is the risk ratio, and lower/upper 95% CI is the 95% confidence interval of the value at risk. **(B)** Survival differences between the high- and low-*POSTN*-expression groups, and patients with a high expression of *POSTN* showed poor overall survival (*p* = 0.039). **(C)** Survival differences between the high- and low-*COL5A2*-expression groups. **(D)** Survival differences between the high- and low-*LPL*-expression groups. **(E)** Survival differences between the high- and low-*VCAN*-expression groups.

To investigate whether the aforementioned 14 DE-ARGs were associated with OS in LUAD patients, we performed a univariate Cox regression analysis in the TCGA-LUAD database. *COL5A2* (*P* = 0.01005), *POSTN* (*P* = 0.01325), *LPL* (*P* = 0.02531), and *VCAN* (*P* = 0.04354) were found to be significantly associated with survival in LUAD patients according to the *P <*0.05 criterion (**Figure 2A**). Furthermore, we tested whether the expression of these 4 genes was correlated with the survival of LUAD patients by K–M survival analysis. The results showed that patients with *POSTN* overexpression had a significantly shorter survival time compared to LUAD patients with relatively low expression levels (*P* = 0.039; **Figure 2B**), whereas the high and low expressions of *COL5A2* (**Figure 2C**), *LPL* (**Figure 2D**), and *VCAN* (**Figure 2E**) were not significantly associated with OS in LUAD patients (all *P >*0.05). The abovementioned evidence implied that inhibition of *POSTN* expression in LUAD patients may be a new indicator for prolonging patient survival. Therefore, we hypothesized that *POSTN* may be a prognostic biomarker for LUAD. However, a single marker is not sufficient to accurately predict the final outcome of patients, so we must pay more attention to the role of *POSTN*-related molecules in disease prognosis.

### POSTN Is Highly Expressed in LUAD and Is Positively Correlated With MVD

**Figure 3:**
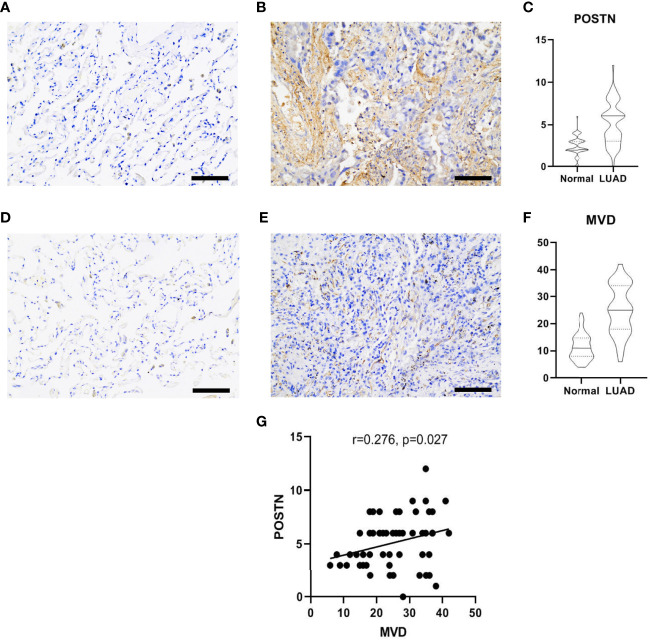
Immunohistochemical staining of lung adenocarcinoma (LUAD) specimens. **(A)** Immunostaining images of POSTN in the adjacent normal tissues. Scale bar = 100 μm. **(B)** The distribution of POSTN in the LUAD tumor tissues in interstitial cells was denser than that in cancer cells. Scale bar = 100 μm. **(C)** The expression of POSTN in tumor tissues was higher than that in the adjacent normal tissues (*p* < 0.0001). **(D)** Immunohistochemical staining of CD34, which was used to mark endothelial cells and to count microvessel density (MVD) in the adjacent normal tissues. Scale bar = 100 μm. **(E)** Distribution of CD34 in the LUAD tumor tissues. Scale bar = 100 μm. **(F)** The expression of MVD in the LUAD tissues was higher than that in the adjacent normal tissues (*p* < 0.0001). **(G)** The correlation analyses revealed that the POSTN expression was positively correlated with MVD in lung adenocarcinoma.

*POSTN* was expressed in the tumor cells and the interstitial cells of LUAD, and the distribution of *POSTN* in the interstitial cells was denser than that in the cancer cells (**Figure 3A, B**), which is consistent to previous studies showing that POSTN is a matricellular protein expressed by stromal cells (22–24). The expression of *POSTN* in the tumor tissue was higher than that in the adjacent normal tissue (**Figure 3C**). The expression of MVD in LUAD was higher than that in the adjacent lung tissue (**Figures 3D–F**), and *POSTN* expression was positively correlated with MVD in LUAD (**Figure 3G**).

### Using WGCNA to Identify the Hub DEGs Related to *POSTN*

**Figure 4:**
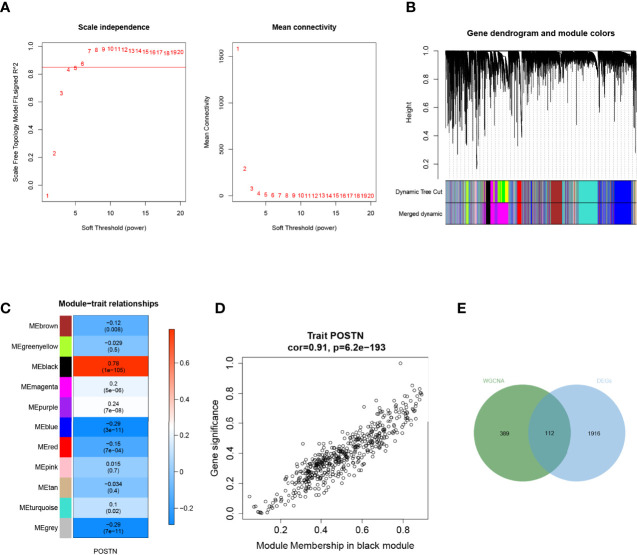
Weighted gene correlation network analysis. **(A)** Soft threshold screening. The horizontal axis of the graph represents the weight parameter power value, and the left vertical axis represents the scale-free fit index, namely, signed *R^2^*, the higher the square of the correlation coefficient, which shows that the network was closer to the scale-free distribution. The vertical axis of the right graph represents the mean of all gene adjacency functions in the corresponding gene module. **(B)** Module merging. Genes were divided into modules through hierarchical clustering, and different colors represent different modules. A total of 11 co-expressed modules were acquired using the dynamic cut-tree algorithm. **(C)** Heat map of the relationship between the module and trait. The black module displayed the highest correlation with *POSTN*. **(D)** Correlation analysis of the gene with module and POSTN in microenvironment black module. **(E)** Screening of POSTN-related differentially expressed genes (DEGs), which yielded a total of 112 genes that were recognized as *POSTN*-related-hub DEGs.

To further characterize the genes associated with *POSTN*, we made use of the “WGCNA” package in the R language. We selected the power *β* = 4 (scale-free *R*^2^ was approximately equal to 0.9) to ensure a scale-free network (**Figure 4A**). After merging similar modules, a total of 11 co-expressed modules were acquired using the dynamic cut-tree algorithm (**Figure 4B**). Furthermore, we performed a correlation analysis between the different co-expression modules and *POSTN* expressive traits. Among the modules, the black module displayed the highest correlation with *POSTN* (correlation coefficient = 0.78, *P* = 1e - 105; **Figure 4C**). Moreover, an intramural analysis of GS and MM of the genes in the black module demonstrated highly meaningful correlations. This finding implied that 510 genes in the black module were significantly associated with *POSTN* (**Figure 4D**). To identify the genes related to both LUAD and *POSTN*, we performed an intersection analysis of the genes in the black module using previously identified DEGs, which yielded a total of 112 genes that were recognized as *POSTN*-related-hub DEGs (**Figure 4E**).

### Functional Enrichment of *POSTN*-Related-Hub DEGs

**Figure 5:**
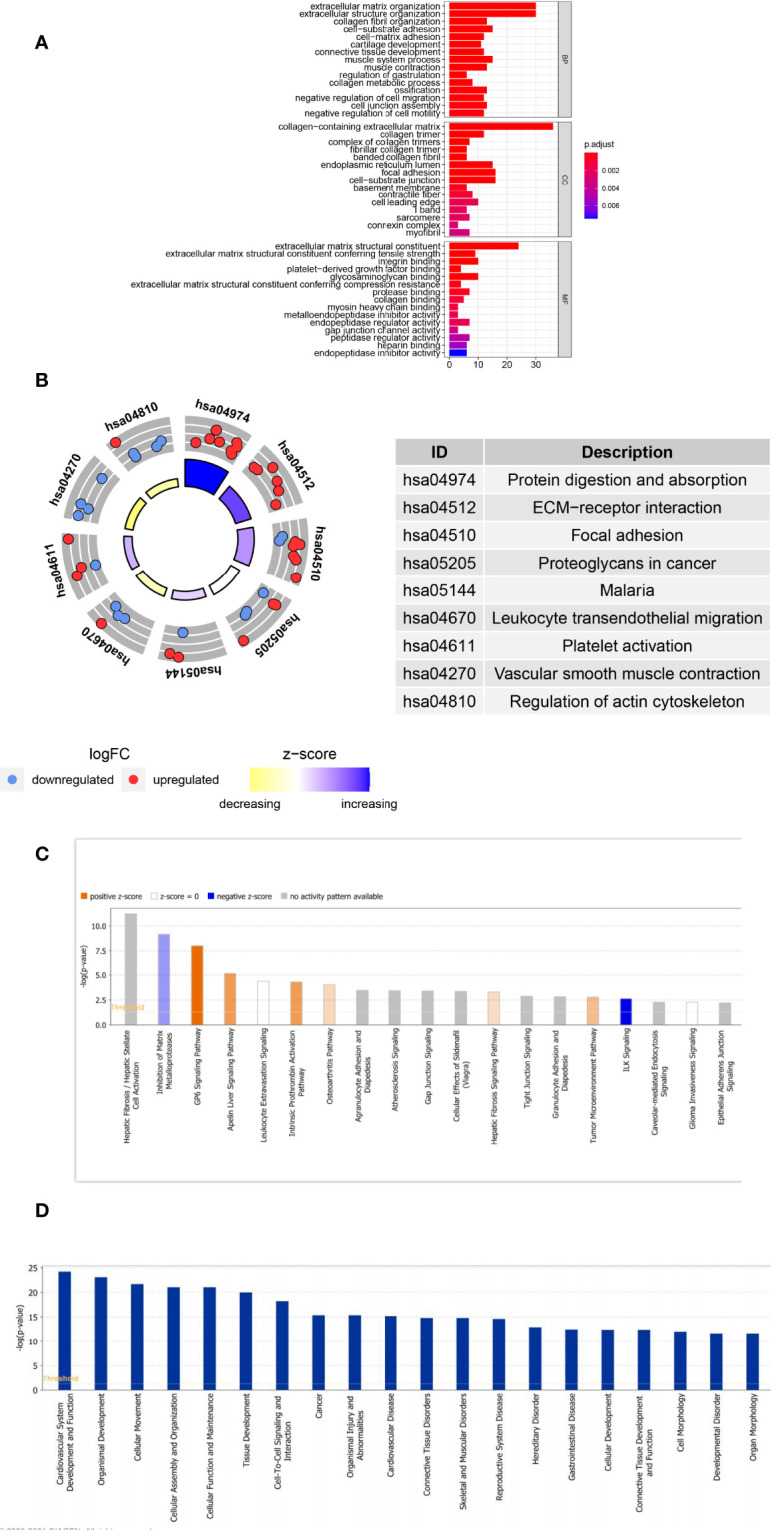
Analyses of the Gene Ontology (GO) and Kyoto Encyclopedia of Genes and Genomes (KEGG) and Ingenuity Pathway Analysis of *POSTN-*related genes. **(A)** GO enrichment analysis. The horizontal axis indicates the number of target genes contained in the GO entry; the vertical axis indicates the name of the GO entry, and the color indicates -log10 *P*-value. **(B)** KEGG enrichment analysis. The z-score was calculated as the number of upregulated genes minus the number of downregulated genes divided by the square root of the number of genes in each pathway. **(C)** The classical pathways of POSTN-related genes were analyzed by IPA. Classical pathways involved a wide variety of signaling pathways, tumor microenvironment, and immune-related pathways. **(D)** Functional pathways of POSTN-related genes analyzed by IPA, which involved disorders of the cardiovascular system, disorders of the connective tissues, cell assembly, movement, and function and maintenance.

GO and KEGG pathway analyses were performed to probe the potential biological functions of *POSTN*-related-hub DEGs. We focused on the BP category. These genes were mainly enriched in terms of the development of the extracellular matrix (ECM) and parenchymal organs. Simultaneously, we found that regulation of vasculature development, artery morphogenesis, artery development, regulation of angiogenesis, aorta morphogenesis, positive regulation of vasculature development, venous blood vessel development, sprouting angiogenesis, aorta development, and positive regulation of blood vessel diameter were prominently enrolled, illustrating the potential involvement of these genes in regulating the pulmonary vascular system during LUAD. Moreover, we observed that epithelial cell-related factors such as proliferation, morphogenesis, and mesenchymal transition were highlighted. Various aspects associated with LUAD pathogenesis, such as connective tissue development, collagen fibril organization, bleb assembly, and gap junction assembly, were also prominent (**Supplementary Table S5**). The top 10 enriched items in the CC and MF categories are exhibited in the middle and bottom panels of **Figure 5A**, and their details are presented in **Supplementary Table S6**. In KEGG analysis, we believed that *POSTN*-related-hub DEGs were most abundant in focal adhesion, leukocyte transendothelial migration, platelet activation, vascular smooth muscle contraction, and regulation of actin cytoskeleton (**Figure 5B**; **Supplementary Table S7**), which supports that these genes were intimately linked to the pathophysiological processes of LUAD.

Furthermore, we employed IPA for canonical pathway and disease and function enrichment analysis of POSTN-related-hub DEGs (**Figures 5C, D**, **Supplementary Tables 8****,** **9**). The outcomes suggested that these genes were primarily engaged in the negative regulation of integrin signaling (ILK signaling) (25), estrogen receptor signaling (26), and inogen receptor signaling (26). On the other hand, the HOTAIR regulatory pathway (27), intrinsic prothrombin activation pathway (28), and GP6 signaling pathway (29) were triggered by these genes as appropriate. Interestingly, all of these canonical pathways, which were either activated or inhibited, had been implicated in the pathological process of lung cancer to varying degrees (**Figure 5C**). We identified these genes as being enriched in a variety of organic diseases (cardiovascular, skeletal and muscular, renal and urological, respiratory, *etc.*), cellular developmental processes, and tumor development (**Figure 5D**).

### Performance of Risk Signature in LUAD

**Figure 6:**
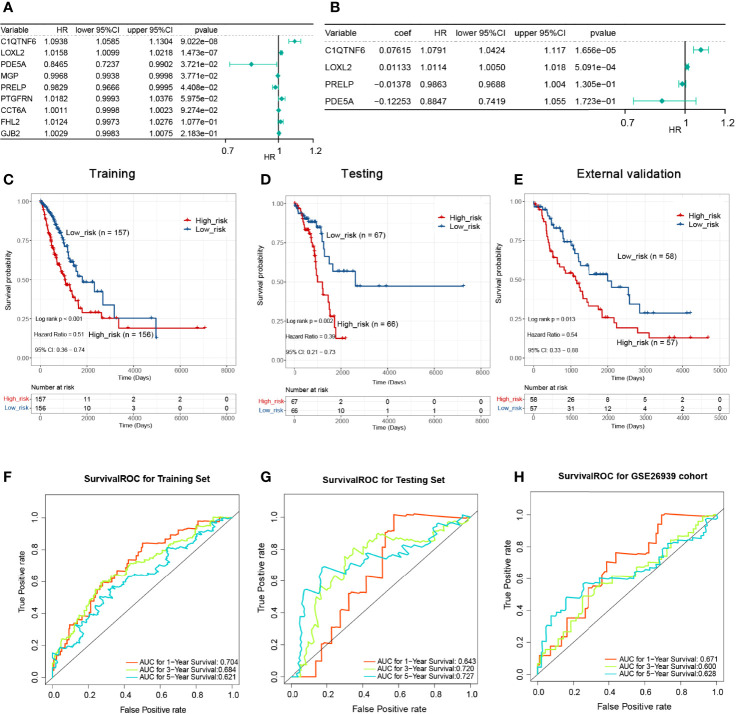
Single-factor and multi-factor Cox regression analysis and construction of proportional hazards model (Cox model). **(A)** Single-factor Cox analysis of the forest map. Five genes with a significant impact on prognosis were determined. **(B)** Multivariate Cox analysis. A prognostic gene signature consisting of four *POSTN*-related-hub differentially expressed genes was established. **(C)** The Kaplan–Meier (KM) survival for the training set with high-/low-risk groups revealed that patients presenting with high-risk scores had a shorter survival time. **(D)** The KM survival for the testing set with a high-/low-risk groups revealed that patients presenting with high-risk scores had a shorter survival time. **(E)** The KM survival for the verification set with a high-/low-risk groups revealed that patients presenting with high-risk scores had a shorter survival time. **(F)** Survival receiver operating characteristic (ROC) for the training set for 1, 3, and 5 years for the risk-based prediction model. **(G)** Survival ROC for the testing set for 1, 3, and 5 years for the risk-based prediction model. **(H)** Survival ROC for the verification set for 1, 3, and 5 years for the risk-based prediction model.

We partitioned the TCGA-LUAD sample randomly into a training set (*n* = 313) and a testing set (*n* = 133) in a 7:3 ratio, whereas the GSE26939 dataset (*n* = 115) was hired as an external validation set in this study. To aid in the development of a valid prognostic status prediction model, K–M analysis was performed along with univariate and multivariate Cox proportional hazard regression analyses to screen for the genes. In the TCGA training set, K–M survival analysis, performed based on the 112 previously identified POSTN-related-hub DEGs, showed that the expression of only 9 genes (*C1QTNF6*, *LOXL2*, *PDE5A*, *MGP*, *PRELP*, *PTGFRN*, *CCT6A*, *FHL2*, and *GJB2*, respectively) was significantly associated with LUAD patients’ OS (*P* < 0.05; **Supplementary Table S10**). Subsequently, we performed a univariate Cox regression analysis on the abovementioned nine genes to further evaluate the effects of these genes on OS in LUAD patients. The results were presented as a forest plot in **Figure 6A**, and *C1QTNF6*, *LOXL2*, *PDE5A*, *MGP*, and *PRELP* were considered as candidate genes for the construction of a prognostic model based on a significance threshold of *P <*0.05. After employing the multivariate Cox proportional hazards regression models, a prognostic gene signature consisting of four *POSTN*-related-hub DEGs, namely, *C1QTNF6*, *LOXL2*, *PDE5A*, and *PRELP5*, was established (**Figure 6B**; **Supplementary Table S11**). Despite the fact that the *P*-values for *PRELP* and *PDE5A* did not satisfy the definition of significance at *P* <0.05, we still incorporated them in the prognostic model for analysis based on the results of the multivariate Cox analysis algorithm and the separate K–M analysis of the four genes mentioned above (**Supplementary Figure S3**).


Risk score=(0.076146× C1QTNF6)+(0.011332× LOXL2)+(−0.12253× PDE5A)+(−0.01378× PRELP)

K–M analyses were performed after grouping by the defined risk scores. The findings revealed that patients presenting with high-risk scores had a shorter survival time in the training set, the testing set, and the external validation set (**Figures 6C–E**). The areas under the ROC curve obtained by predicting patient survival at 1, 3, and 5 years were 0.704, 0.684, and 0.621 for the risk-based prediction model in the training set (**Figure 6F**), 0.643, 0.720, and 0.727 in the testing set (**Figure 6G**), and 0.671, 0.600, and 0.628 in the external validation set, respectively (**Figure 6H**).

**Figure 7:**
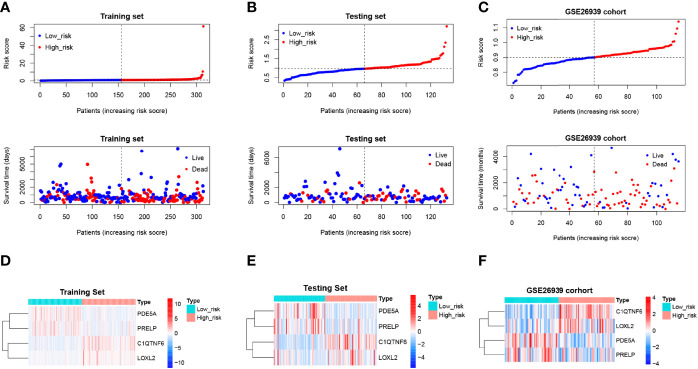
Risk profile of the risk model and heat map of gene expression in the high-risk and low-risk groups. **(A–C)** Training, test, and validation set risk curves. Scatter plots were derived from survival times and risk scores. With increasing risk scores, the number of patients who died increased, and the survival gradually shrank. **(D–F)** Training, test, and validation set model gene expression heat maps. The horizontal axis represents the sample, while the horizontal axis represents the risk score and the survival of the sample. The vertical reference line represents the sample position corresponding to the median risk score, with low-risk patients on the left and high-risk patients on the right. According to the heat map results of the training, test, and verification sets, PDE5A and PRELP were poorly expressed in the high-risk group, while C1QTNF6 and Loxl2 were highly expressed in the high-risk group.

Scatter plots were derived from survival times and risk scores. With increasing risk scores, the number of patients who died increased, and the survival gradually shrank in the training (**Figure 7A**), testing (**Figure 7B**), and external validation sets (**Figure 7C**). However, the expression levels of *C1QTNF6* and *LOXL2* increased in the high-risk group (**Figures 7D–F**), which suggests that the abnormal expression of these genes was involved in the adverse prognosis of LUAD.

In addition, IPA revealed an interplay network of four prognostic genes (**Supplementary Figure S4**). Except for *C1QTNF6* and *LOXL2*, which had a complex indirect relationship, the remaining prognostic genes possessed their autonomous networks of action. Interestingly, *LOXL2* was predicted to be directly targeted by *POSTN*, although this result was not experimentally validated.

### The Risk Score as an Independent Prognostic Factor

**Figure 8:**
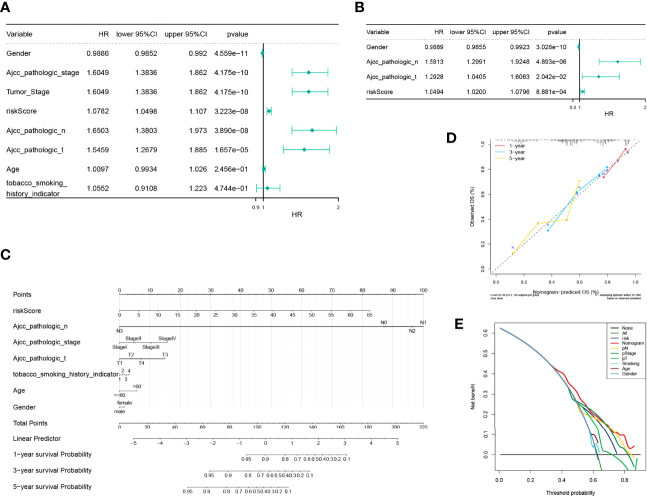
Independent prognostic analysis of the risk model and construction and verification of the survival prediction sequence diagram. **(A)** An independent prognosis of univariate Cox analysis showed that risk score, sex, American Joint Committee on Cancer (AJCC) pathologic stage, tumor stage, AJCC pathologic N stage, and AJCC pathologic T stage had a prognostic value for overall survival in lung adenocarcinoma. **(B)** Independent prognosis of multivariate Cox analysis, risk score, and gender, and pathological N and T staging acted as independent prognostic factors. **(C)** Line chart of the clinical factors; these variables are visualized in the nomogram. In tobacco smoking history indicator, 1 represents lifelong non-smoker (<100 cigarettes smoked in a lifetime), 2 represents current smoker (includes daily smokers non-daily/occasional smokers), 3 represents current reformed smoker for >15 years, 4 represents current reformed smoker for ≤15 years. **(D)** Line chart 1-, 3-, and 5-year calibration curve. The calibration curves indicated that the nomogram exhibited a satisfactory agreement between the predicted and observed values for 1-, 3-, and 5-year survival rates. **(E)** The 5-year decision-making curves of the line chart and other clinical traits are presented. The abscissa indicates the threshold probability. In the risk assessment tool, the probability of patient i being diagnosed with the disease is recorded as Pi; when Pi reaches a certain threshold (recorded as Pt), it is defined as positive, and treatment is initiated. There are patient-treated benefits (regarded as benefits) as well as patient-treated injuries and untreated losses (regarded as disadvantages). The ordinate is the net benefit, where the pros minus the cons. Different oblique lines in the chart represent different clinical diagnostic models.

Consequent analyses demonstrated that the risk scores were substantially correlated with multiple clinical traits, such as age, AJCC pathologic stage, AJCC pathologic TNM stage, tumor stage, and smoking habit (**Supplementary Figure S5**). Moreover, a stratified survival analysis indicated that our risk scoring system could predict the prognosis of patients under almost all clinical traits (**Supplementary Figure S6**). To estimate the independent prognostic validity of the signature, univariate and multivariate Cox proportion hazard regression models were applied (**Figures 8A, B**). The results from the univariate analysis showed that risk score (HR = 1.0782, *P* < 0.001), sex (HR = 0.9886, *P* < 0.001), AJCC pathologic stage (HR = 1.6049, *P* < 0.001), tumor stage (HR = 1.6049, *P* < 0.001), AJCC pathologic N stage (HR = 1.6503, *P* < 0.001), and AJCC pathologic T stage (HR = 1.5459, *P* < 0.001) had a prognostic value for OS in LUAD. In multivariate Cox regression analysis, risk score (HR = 1.0494, *P* = 8.881e - 04), sex (HR = 0.9889, *P* = 3.028e - 10), AJCC pathology N stage (HR = 1.5813, *P* = 4.893e - 06), and AJCC pathology T stage (HR = 1.2928, *P* = 2.042e - 02) retained their prognostic value. These variables were visualized in the nomogram (**Figure 8C**), and the calibration curves indicated that the nomogram exhibited a satisfactory agreement between the predicted and observed values for 1-, 3-, and 5-year survival rates (**Figure 8D**). In addition, the consequences of the DCA indicated that the nomogram was likely to provide a potentially higher clinical benefit (**Figure 8E**).

### Analysis of Immune Cell Infiltration Between the High- and Low-Risk Groups

**Figure 9:**
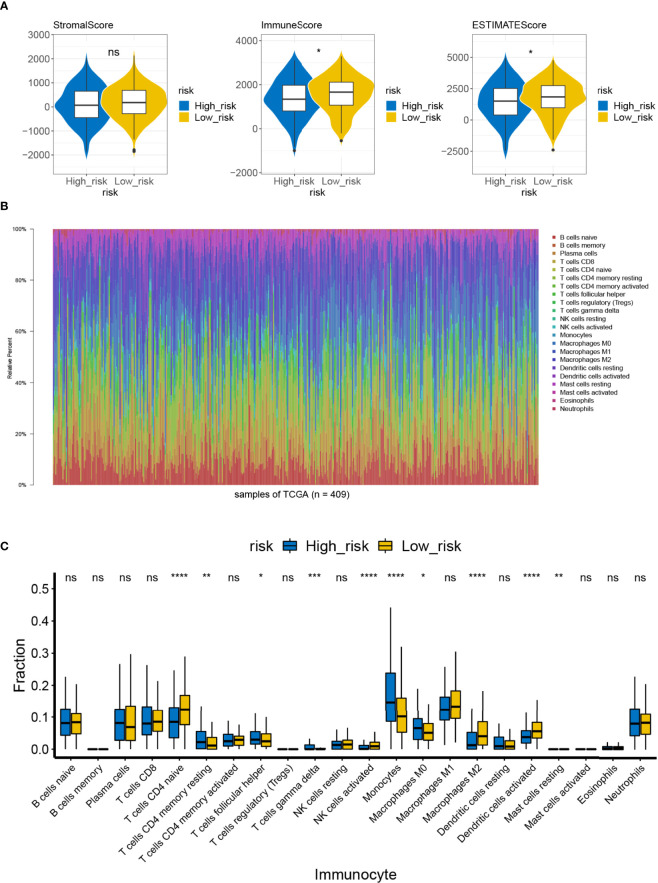
Difference in immune infiltration and microenvironment between the high- and low-risk groups. **(A)** The immune microenvironment of the high-/low-risk groups was analyzed by ESTIMATE, and the immune score and the ESTIMATE score of the high-/low-risk groups were lower ns, not significant. **(B)** Abundance ratio of immune cells in the 446 LUAD cases. Each column represents a sample, and each column with a different color and height indicates the abundance ratios of immune cells in this sample. **(C)** A rank-sum test was performed to analyze the difference in the immune cells between the high- and low-risk groups. Significant differences were noted in the 10 immune cells between the high- and low-risk groups; T-cells CD4 naive, activated NK cells, M2 macrophages, and activated dendritic cells in the high-risk groups showed lower immunological infiltrations. ns, not significant. *P<0.05, **P<0.01, ***P<0.001, ****P<0.0001.

By reviewing the results of previous functional enrichment analyses, we found that prognostic genes were present in several immune-related pathways and diseases. Therefore, we further investigated the relationship between the risk score and the immune microenvironment. We first analyzed the differences in immune scores, stromal scores, and ESTIMATE scores between the high- and low-risk groups within the ESTIMATE algorithm. We learned that the immune and ESTIMATE scores were significantly higher in the low-risk group than in the high-risk group (**Figure 9A**). Furthermore, CIBERSORT had analyzed the immune cell infiltration in all patient samples meeting the criterion of *P* <0.05 in the TCGA-LUAD (*n* = 446) (**Figure 9B**). A total of four types of infiltrating immune cells were detected as being less infiltrated in the high-risk group, namely, naïve CD4 T cells, activated NK cells, M2 macrophages, and activated dendritic cells. On the other hand, resting memory CD4 T cells, follicular helper T cells, gamma delta T cells, monocytes, M0 macrophages, and resting mast cells in the high-risk group had high infiltration levels (**Figure 9C**). Such evidence suggests that the risk scoring system may influence patient prognosis by altering the degree of infiltration of these immune cells.

### Construction of ceRNA Network Based on Four Prognostic Genes

**Figure 10:**
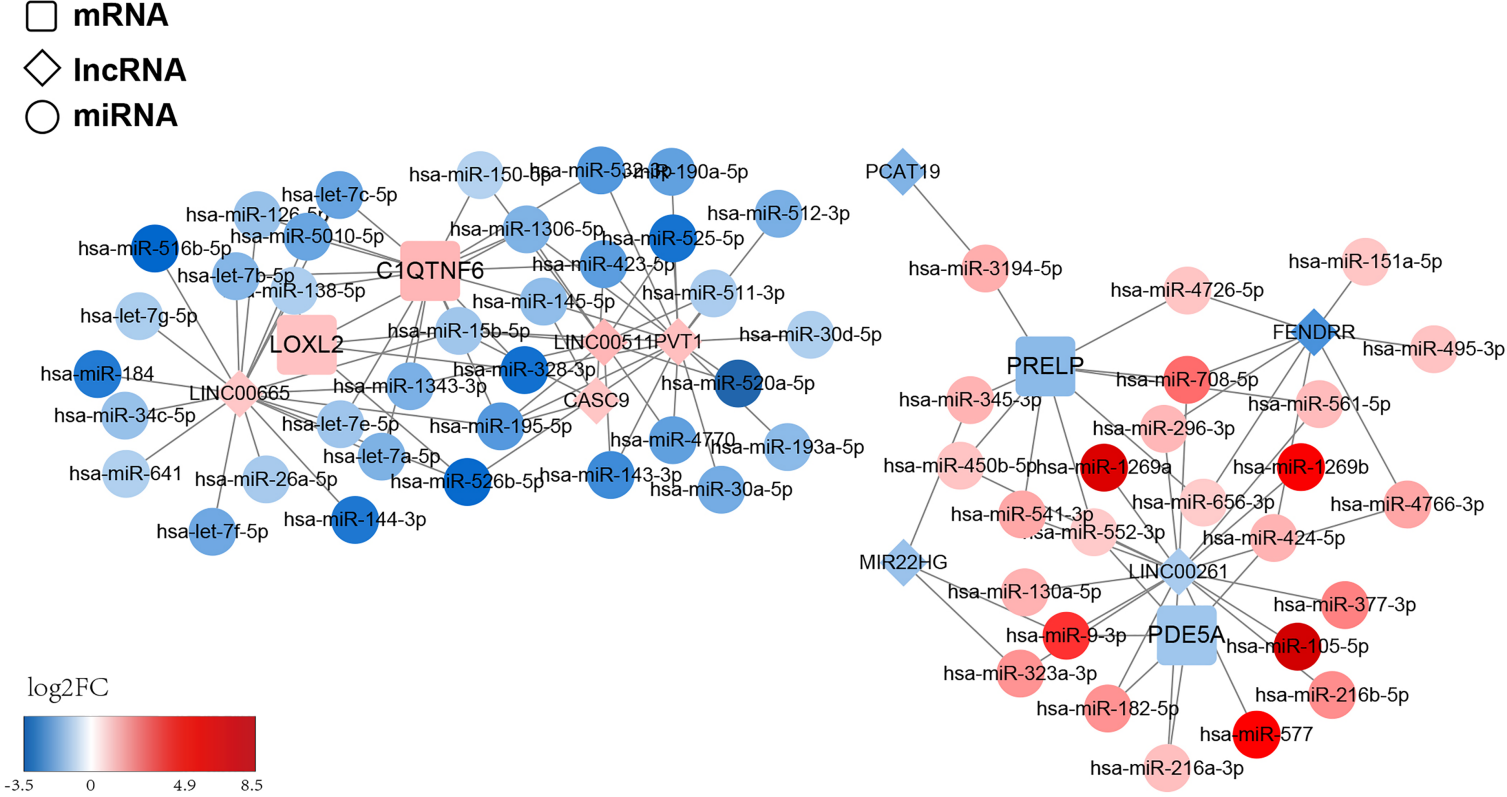
ceRNA regulatory network of the risk model gene. The squares represent model genes, the diamonds represent lncRNA, and the circles represent microRNAs. Different colors represent different log_2_FC values. We predict the prognostic gene–DE-miRNAs–DE-lncRNAs relationship pairs and obtained a ceRNA network based on 4 prognostic genes, which covered 72 nodes and 116 edges.

To structure the ceRNA network, we obtained 428 LUAD-related DE-miRNAs from the TCGA-LUAD database. Meanwhile, we sifted 43 DE-lncRNAs based on the 2,028 DEGs previously obtained. Subsequently, we utilized the starBase database and miRanda software to predict the prognostic gene–DE-miRNAs–DE-lncRNAs relationship pairs. We finally obtained a ceRNA network based on four prognostic genes that covered 72 nodes and 116 edges (**Figure 10**). Specifically, four lncRNAs (*LINC00665*, *PVT1*, *LINC00511*, and *CASC9*) could competitively bind to 10 miRNAs and upregulate *C1QTNF6* expression. Moreover, the abovementioned lncRNAs could regulate the expression of *LOXL2* by binding to five miRNAs. Additionally, *PDE5A* could be regulated by three lncRNAs (*LINC00261*, *MIR22HG*, and *FENDRR*) through the competitive binding of five miRNAs. lncRNA *PCAT19* and the abovementioned three lncRNAs could also target nine miRNAs and cause the downregulation of *PRELP* expression. We found that the competitive binding of lncRNA *LINC00261* to *hsa-miR-552-3p* could simultaneously downregulate the expression of *PDE5A* and *PRELP*. This information is provided in **Supplementary Table S12**.

## Discussion

LUAD is the most common type of non-small cell lung cancer with a poor prognosis. The progression of LUAD depends on the formation of new blood vessels, which provides nutrients for tumor cells and facilitates the removal of metabolites. Angiogenesis is critical for tumor growth, invasion, and metastasis (30, 31). Tumor angiogenesis is closely related to the release of angiogenic factors by the tumor cells and the changes in TME, and the interactions between TME and tumor cells are essential for angiogenesis. The TME can be altered by the tumor cells, and the TME can, in turn, affect the biological behavior of the tumor cells (32). Changes in the stroma of cancer are accompanied by the differentiation of fibroblasts into cancer-associated fibroblasts, which alter the ECM component in the tumor region and promote the formation, angiogenesis, and metastasis of epithelial tumors (33).

ECM plays an indispensable role in the “dialogue” between the tumor cells and the components of their microenvironment. ECM consists of structural and non-structural proteins with various functions. The matrix proteins can bind to other ECM proteins, growth factors, and cytokines *via* cell-specific membrane surface receptors (34). The matrix proteins regulate cell proliferation and differentiation, which maintains homeostasis of normal tissues (32). These proteins include thrombospondin, osteopontin, members of the *CCN* family (*Cyr61*, *CCN2*, and *CCN3*), *SPARC*, fibular proteins, and periostin (*POSTN*) (35). In the present study, we analyzed the angiogenic datasets associated with LUAD and found seven significantly upregulated datasets and seven significantly downregulated datasets. Three DE-ARGs (*COL5A2*, *POSTN*, and *VCAN*) acted as risk factors, and *LPL* functioned as a protective factor for patients with LUAD according to the univariate Cox regression models. *COL5A2* is an important component of blood vessels and maintains their tension and elasticity (36). *LPL* is a lipid metabolism gene and a risk factor for arteriosclerosis (37). *VCAN* is involved in tumor proliferation, migration and angiogenesis, which plays an important role in histomorphology (38). In our study, K-M analysis showed that patients with overexpression of *POSTN* had significantly shorter survival time, while the expression of COL5A2, *VCAN*, and LPL did not significantly differentiate the prognosis of LUAD patients. Therefore, the low expression of *POSTN* may serve as a new indicator of long-term survival in LUAD patients, and POSTN may be regarded as a prognostic biomarker in LUAD.

As an ECM protein, *POSTN* promotes the invasion and metastasis of several tumors. The immortal proliferative ability and reduced intercellular adhesion of tumor cells make it easier to infiltrate into tissues, induce angiogenesis, and evade immune surveillance, thereby acquiring invasive and metastatic potential. And *POSTN* is found to play an important role in promoting tumor cells acquiring those properties (39, 40). *POSTN* regulates the proliferation, angiogenesis, invasion, and metastasis of cancer cells by interacting with integrins (41, 42). *POSTN* is overexpressed in many human cancers, including ovarian cancer, head and neck cancer, and pancreatic ductal cancer (43–45). Studies have reported the effect of POSTN expression on the prognosis of non-small cell lung cancer (46). *POSTN* has been shown to promote angiogenesis in malignant tumors. The proliferation ability of breast cancer cells with *POSTN* expression was significantly enhanced, and the tumor formation test revealed that the vessel density was significantly increased (40). This study also demonstrated that POSTN could improve angiogenesis by upregulating the expression of Flk-1/KDR, which is the receptor of *VEGF*. Studies on colon cancer have suggested that *POSTN* promotes angiogenesis during the development of liver metastases (39). A study on osteosarcoma has implied that *POSTN* overexpression is associated with tumor angiogenesis and poor prognosis. A positive correlation was seen between the expression of *POSTN* and *VEGF*, and the microvessel density in case of an elevated expression of *POSTN* was significantly higher than that of normal bone tissue. A multivariate analysis confirmed that *POSTN* overexpression was an independent risk factor for prognosis in patients with osteosarcoma (47).

Tumor angiogenesis involves multiple growth factors and signal transduction pathways, and the heterogeneity of tumors with different tissue sources and varying degrees of differentiation complicates the regulation of angiogenesis (48). At present, several signal pathways such as VEGF/VEGFR, PI3 K-Akt, Notch signal pathway, Ang-Tie signal pathway, and PDGF/PDGFR have been observed to be involved in tumor angiogenesis (49–51). In our study, we used WGCNA to assess the genes related to *POSTN*. Furthermore, we employed GO and KEGG pathway analyses to probe the potential biological functions of *POSTN*-related-hub DEGs. These genes were mainly enriched in terms of the development of the ECM and the parenchymal organs. We identified the genes that may be involved in the regulation of angiogenesis in LUAD. These genes play important roles in vasculature development, morphogenesis of blood vessels, regulation of angiogenesis, sprouting angiogenesis, and positive regulation of blood vessel diameter. In addition, gene enrichments associated with the pathogenesis of LUAD were observed, which were linked to connective tissue development, collagenous tissue, bleb assembly, and gap junction assembly. *POSTN* has been documented to be related to the properties of decrease in intercellular adhesion, induction of angiogenesis, avoidance of immune surveillance, and metastasis potential of tumor cells (23, 52–54). In our study, *POSTN*-related-hub DEGs were found to be involved in local cell adhesion, leukocyte–endothelial turnover, platelet activation, vascular smooth muscle contraction, and actin cytoskeleton regulation. These genes have been reported to be associated with three important regulators and receptors of tumor angiogenesis, namely, VEGF, fibroblast growth factor, and platelet-derived growth factor (55, 56), which suggests that these genes are closely related to the pathophysiological process of LUAD. We also found that the epithelial cell-related genes, such as those involved in cell proliferation, morphogenesis, and mesenchymal changes, were enriched. These results signify the presence of extensive connections between the tumor cells and the tumor stroma in the pathophysiological process of LUAD, especially in tumor angiogenesis and its regulation. In the typical pathway and disease function related to *POSTN* enrichment analysis, integrin signaling (ILK signaling), estrogen receptor signaling, and inogen receptor signaling were found. These pathways are associated with cell adhesion and changes in the microenvironment of tumor stromal cells, and they ultimately promote tumor progression. These genes play a crucial role in many organ diseases, cell differentiation, and tumor development (57, 58).

The body’s immune surveillance system plays a key role in the identification, killing, and elimination of mutant cells (tumor cells). Tumors develop when the body’s normal immune system is suppressed and the immune surveillance function is weakened. In the TME, immunosuppressive cells such as Treg, TAMs, and MDSCs are often present. Furthermore, accumulation of IL-10, deficiency of perforin and granzyme secreted by CD8^+^ T cells, a decrease of cytotoxicity, and inactivation of natural killer cells are observed (59, 60). Studies have found that patients who do not respond well to anti-angiogenic therapy experience an increase in immunosuppressive cells in the TME; hence, immunosuppression in the TME is not conducive to the prognosis of antiangiogenic therapy (61). We have identified prognostic genes in several immune-related pathways and diseases in our present study. We have further investigated the relationship between the risk score and the immune microenvironment. In the high-risk group, four types of infiltrating immune cells with low expression were detected, namely, naive CD4 T cells, activated NK cells, M2 macrophages, and activated dendritic cells. The extent of immune cell infiltration affects the prognosis of patients with LUAD. As a gene with survivorship differences, POSTN can induce the accumulation of tumor-associated macrophages around the tumor cells and may play a supportive role for TAMs in tumor progression (52). Distinct immune cell clusters with macrophage predominance characterize an aggressive hepatocellular carcinoma phenotype, defined molecularly by angiogenic gene enrichment and clinically by poor prognosis (62). *POSTN* promotes the accumulation of myeloid inhibitory cells in the lung (63). In the absence of *POSTN*, the immunosuppressive function of these cells is reduced, which affects tumor progression. The mechanisms of immune escape are diverse, and the function of *POSTN* in these mechanisms has not been studied until recently. Therefore, it is worthwhile to examine the role of POSTN in the immune escape mechanism.

Transcription elements, such as lncRNA and mRNA, compete for binding to miRNA through the miRNA response element and then regulate each other’s functions (64, 65). Interference with the ceRNA may affect carcinogenesis or other complex diseases, thus, the identification of these ceRNAs will allow us to better understand the pathogenesis and development of cancers (66). To explore the regulatory pathways related to prognostic genes, a ceRNA network based on the four prognostic genes was constructed. Specifically, lncRNAs (LINC00665, PVT1, LINC00511, and CASC9) could regulate the expression of *LOXL2* by binding to five miRNAs, whereas LOXL2 could directly interact with *POSTN* in the ceRNA network.

Therefore, we expect that miRNA, mRNA, and lncRNA in this regulatory network may interact with each other in tumor vascular regulation. Hence, detecting their expression levels may provide a new target for early-stage LUAD prediction and a new method of non-invasive diagnosis.

The angiogenetic factors and regulatory pathways involved in tumor angiogenesis are quite complex. Previous studies have focused on specific single regulatory genes, and the full range of genes and pathways related to tumor angiogenesis is not well understood. Fortunately, owing to the advances in artificial intelligence and the rise of the big data era, it is possible to grasp a much broader range of information from the database. In our study, we used bioinformatics tools to screen the vast amount of biological information about tumor angiogenesis, which made it possible to solve the difficult problem of sorting the data from complicated biological signaling pathways. Therefore, we expect that miRNA, mRNA, and lncRNA, in this regulatory network, may interact with each other in tumor vascular regulation. However, our research, which focused on the tumor vascular biology of LUAD, is limited by the number of available cases. We also did not specifically analyze the expression of these genes in early-stage LUAD alone, nor did we analyze the impact of these genes on the survival and diagnosis of LUAD, so prediction with those genes may cause a bias in early-stage lung cancer. Moreover, this study is a retrospective study, and further research is needed to determine whether we can prospectively predict tumorigenesis and angiogenesis before LUAD diagnosis in the general population.

## Conclusion

In this study, we examined genes associated with angiogenesis in LUAD by bioinformatics analysis and demonstrated that *POSTN* is one of the key genes regulating angiogenesis. In addition, we constructed a new prognostic gene signature associated with ARGs for the prognosis of LUAD patients. We further analyzed the relationship between the risk score and the immune microenvironment, and established a ceRNA interaction network for further study of tumor angiogenesis in LUAD. However, the study of *POSTN* expression in LUAD, especially in the early stage of the disease, is insufficient. In addition, the expression and function of *POSTN* in stromal cells and the communication between *POSTN* and cancer cells are yet to be elucidated. The role of *POSTN* in angiogenesis also needs to be further explored.

## Data Availability Statement

Publicly available datasets were analyzed in this study. This data can be found here: The Cancer Genome Atlas (TCGA) https://www.cancer.gov/about-nci/organization/ccg/research/structural-genomics/tcga.

## Ethics Statement

This study was approved by the Ethics Committee of The First Affiliated Hospital of Shandong First Medical University and Shandong Province Qianfoshan Hospital. The patients/participants provided their written informed consent to participate in this study.

## Author Contributions

DS reports that he contributed to the design of the study, acquisition of the data, analysis of the data, interpretation of results, and critical review of the manuscript. ZG reports that he contributed to the analysis of the data and critical review of the manuscript. JW reports that he contributed to the immunohistochemical staining and analysis of the data. QC reports that he contributed to the acquisition and analysis of the data. All authors contributed to the article and approved the submitted version.

## Funding

This work was supported by the Science and Technology Development Plan of Jinan (to DS, 201907061) and the Natural Science Foundation of Shandong Province (to QC, ZR2020KH026).

## Conflict of Interest

The authors declare that the research was conducted in the absence of any commercial or financial relationships that could be construed as a potential conflict of interest.

## Publisher’s Note

All claims expressed in this article are solely those of the authors and do not necessarily represent those of their affiliated organizations, or those of the publisher, the editors and the reviewers. Any product that may be evaluated in this article, or claim that may be made by its manufacturer, is not guaranteed or endorsed by the publisher.

## Supplementary Material

Supplementary Table 1 HALLMARK_ANGIOGENESIS gene set, where the 36 angiogenesis-related genes were obtained.

Supplementary Table 2 For the miRNAs in the network, there were 428 DE-miRNAs, of which 251 were up-regulated and 177 were down-regulated genes.

Supplementary Table 3 Forty-three DE-lncRNAs identified from 2028 DEGs were exhibited.

Supplementary Table 4 Forty-three A total of 2028 DEGs, including 924 up-regulated genes and 1104 down-regulated genes, were appraised compared to normal tissues.

Supplementary Table 5 Various terms were associated with LUAD pathogenesis, such as connective tissue development, collagen fibril organization, bleb assembly, and gap junction assembly were exhibited.

Supplementary Table 6 The enriched items in the CC and MF categories were also exhibited and their details were consolidated.

Supplementary Table 7 In KEGG analysis, POSTN-related-hub DEGs were most abundant in Focal adhesion, Leukocyte transendothelial migration, Platelet activation, Vascular smooth muscle contraction, and Regulation of actin cytoskeleton.

Supplementary Table 8 IPA was employed for canonical pathway enrichment analysis of POSTN-related-hub DEGs.

Supplementary Table 9 IPA was employed for disease & function enrichment analysis of POSTN-related-hub DEGs.

Supplementary Table 10 Nine genes with positive effects on the OS in the training set by K-M analysis.

Supplementary Table 11 Using multivariate Cox proportional hazards regression models, a prognostic gene signature was established consisting of four POSTN-related-hub DEGs.

Supplementary Table 12 Competitive binding of lncRNA LINC00261 to hsa-miR-552-3p could be simultaneously down-regulated the expression of PDE5A and PRELP. The explicit information was integrated.
