# Supplementary material for: Prognostic Impact of the Angiogenic Gene POSTN and Its Related Genes on Lung Adenocarcinoma
Source: Front Oncol. 2022 Jun 27;12:699824. doi: 10.3389/fonc.2022.699824 (PMC9271775; doi:10.3389/fonc.2022.699824)
Supplement: Supplementary file 13 [file DataSheet_1.docx]

**Figure S1. WGCNA analysis**


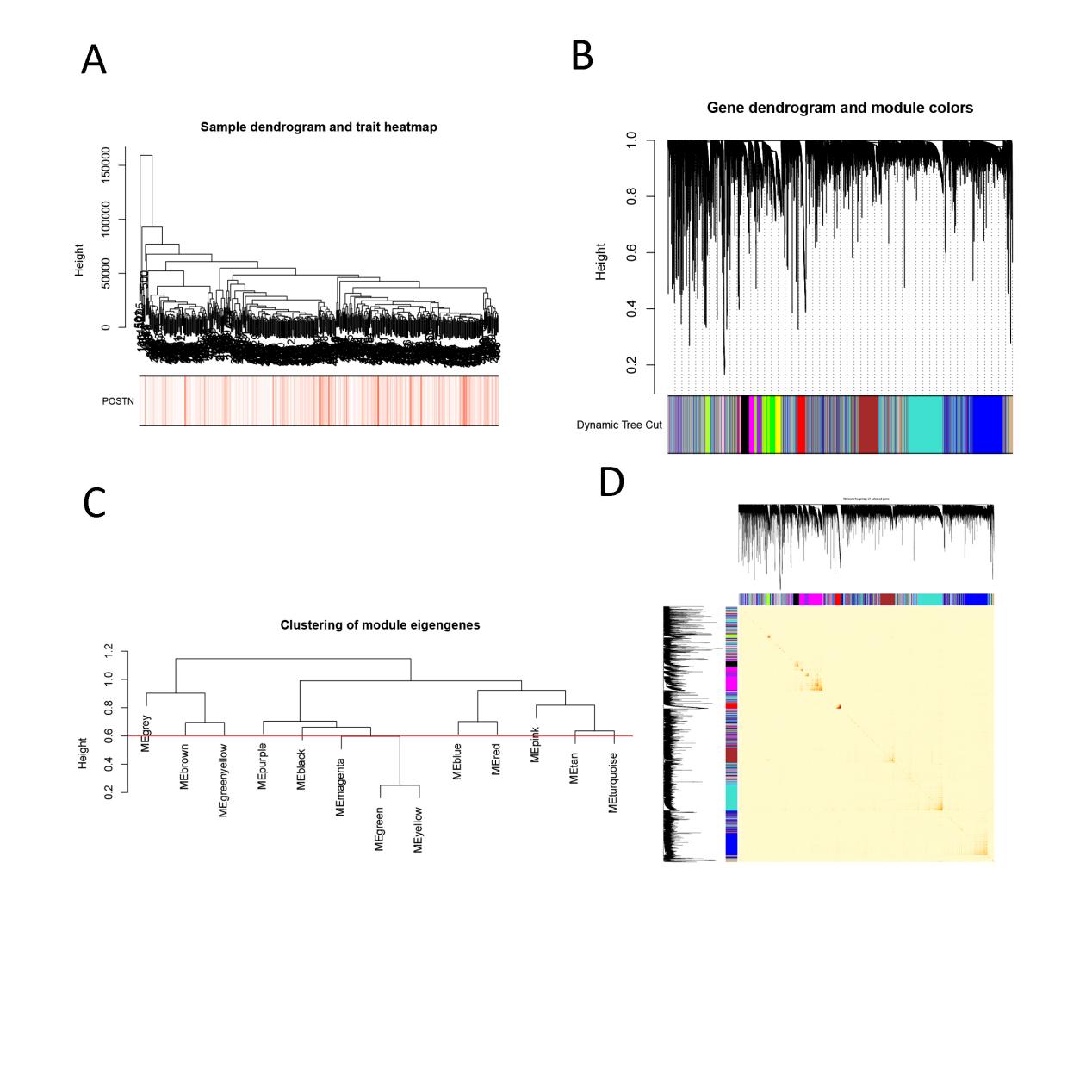


1. Sample clustering. The branches represent the sample, and the ordinate represents the height of the hierarchical cluster. There is no abnormal outlier in the sample, so no need to delete the sample.
2. Module partition using dynamic tree cutting method. Genes are divided into modules by hierarchical clustering, and different colors represent different modules.
3. Clustering of module eigengenes: module identification.
4. WGCNA heatmap.

**Figure S2. ceRNA regulatory network of risk model genes.**


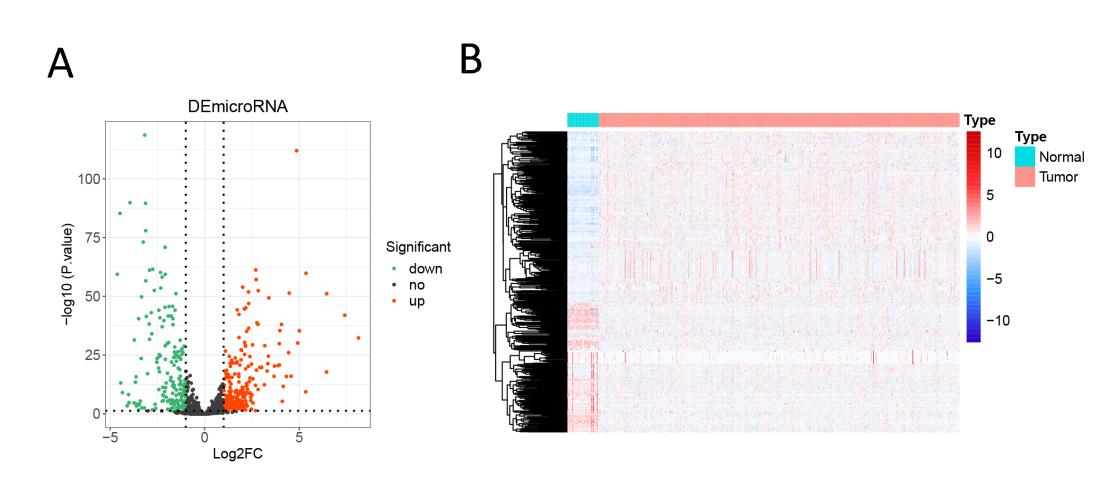


1. Differential microRNA volcano map. We analyzed 428 microRNAs from Lung adenocarcinoma patients with TCGA, including 251 microRNAs with up-regulation and 177 microRNAs with down-regulation.
2. Differential heatmap of microRNA expression.

**Figure S3. Assessing the prognostic value of risk models.**


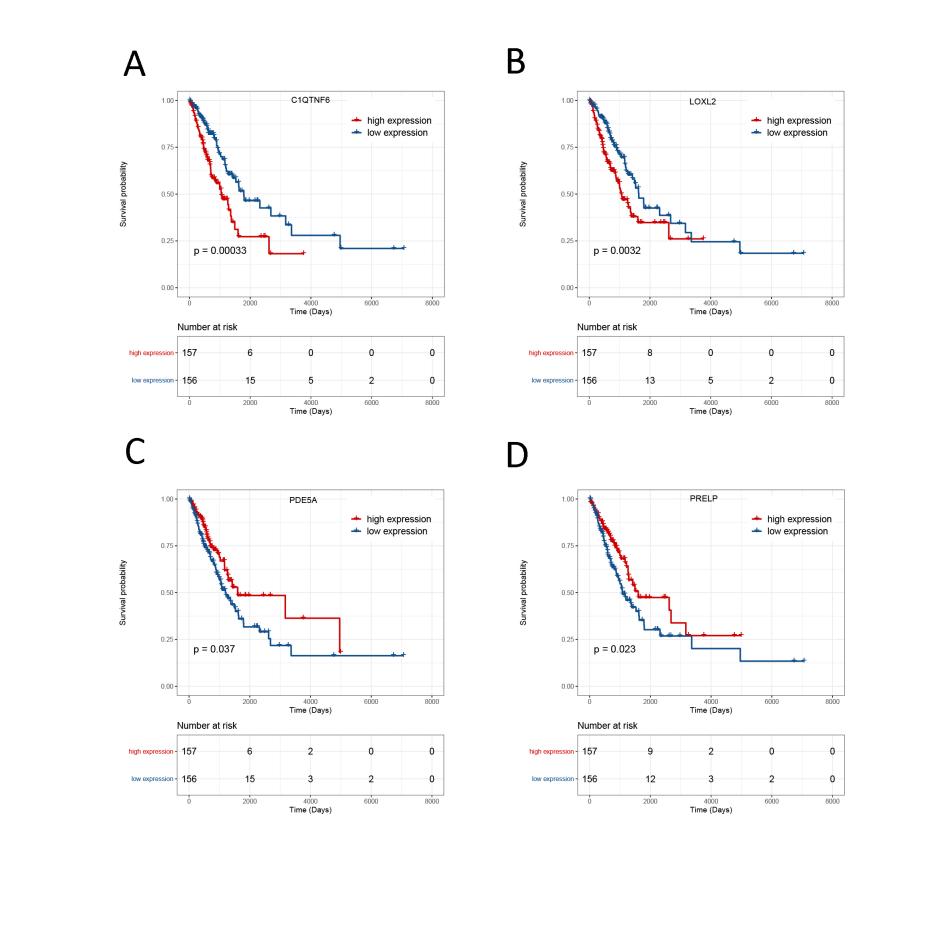


The KM survival curve of the four model genes(A: C1QTNF6; B: LOXL2; C: PDE5A; D: PRELP) in the training set. The abscissa is survival time and the ordinate is survival rate. The expression of 4 model genes was significantly correlated with survival time.

**Figure S4. Prediction of risk model gene with IPA gene interaction network**


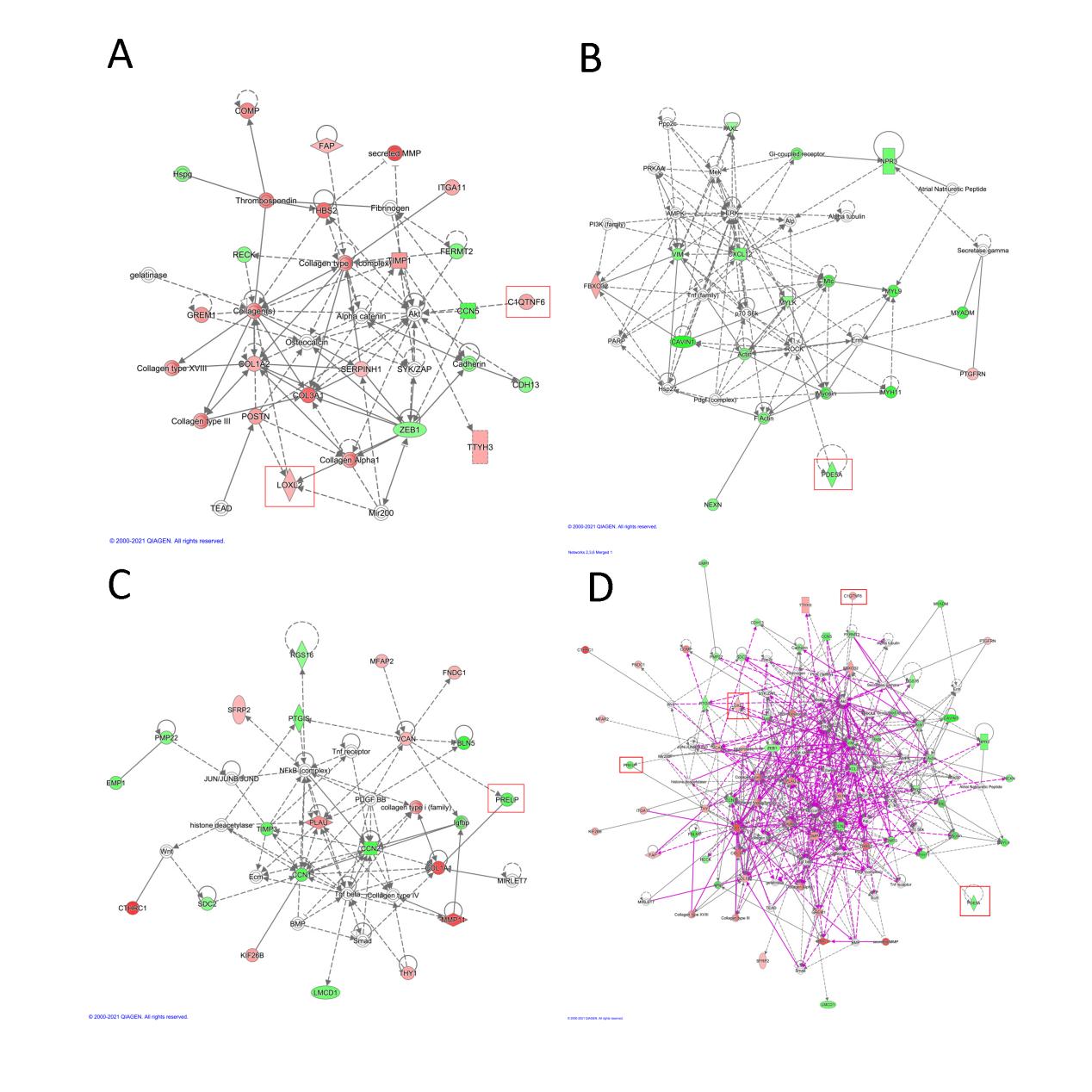


1. C1QTNF6 and LOXL2 were in the same network, in which LOXL2 was predicted a direct interaction with POSTN. Dotted lines in a network diagram represent interactions that have not been experimentally validated, while straight lines represent experimentally validated interactions.
2. Prediction of the gene interaction network of PDE5A by IPA,
3. Prediction of the gene interaction network of PRELP by IPA. The Red and green color represent the up-regulated and down-regulated genes of the POSTN-related differentially expressed genes, and the gray color represents the predictive genes. The model genes are circled in red. The different shapes of the circled genes represent different gene types.
4. Model Gene Network merging diagram.

**Figure S5. Analysis of correlation between risk score and clinical features**


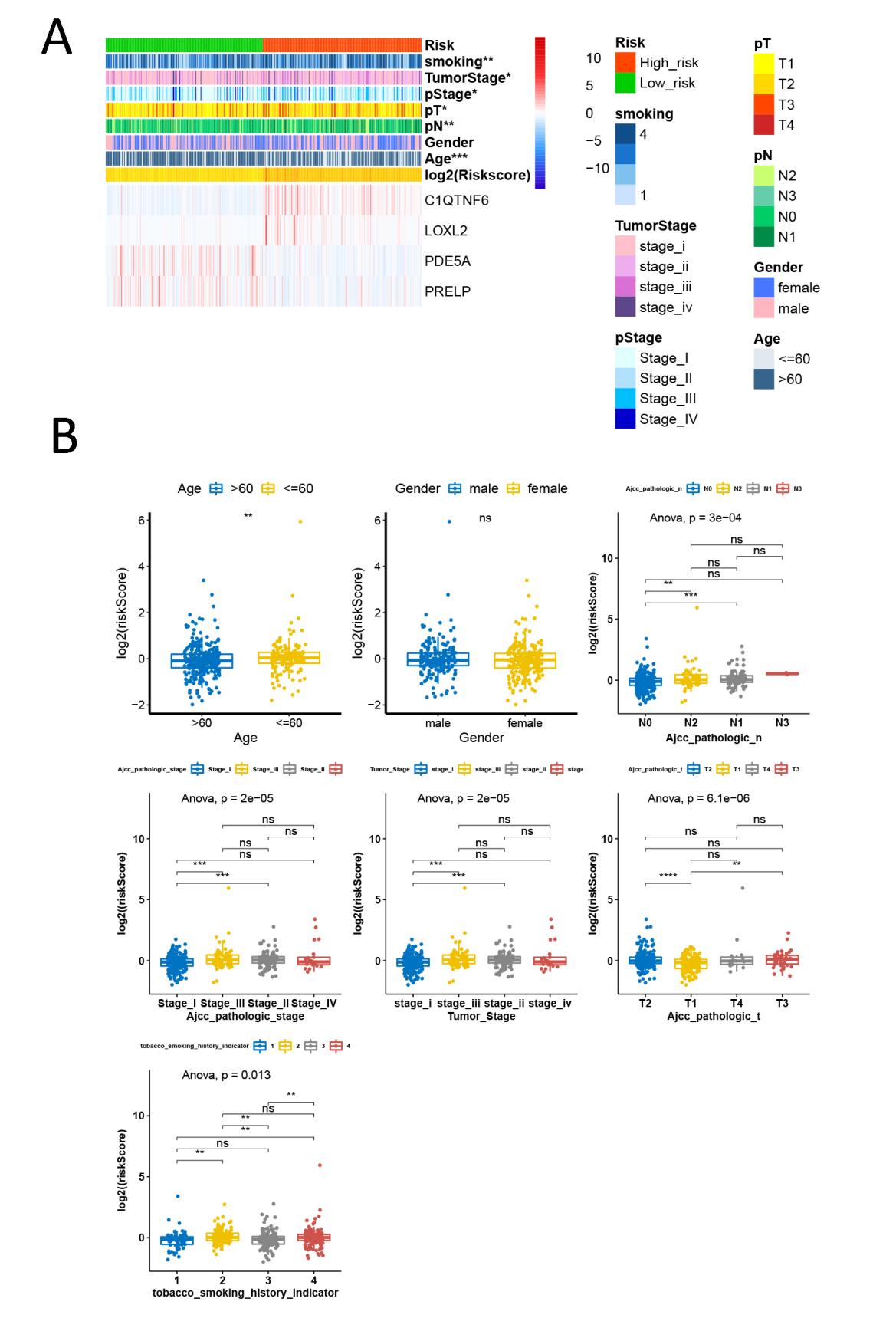


1. Heatmap of model genes and clinical traits. The results showed that all clinical traits except gender were associated with risk score.
2. Correlation between risk score and clinical traits.

**Figure S6. Stratified survival analysis of risk model**


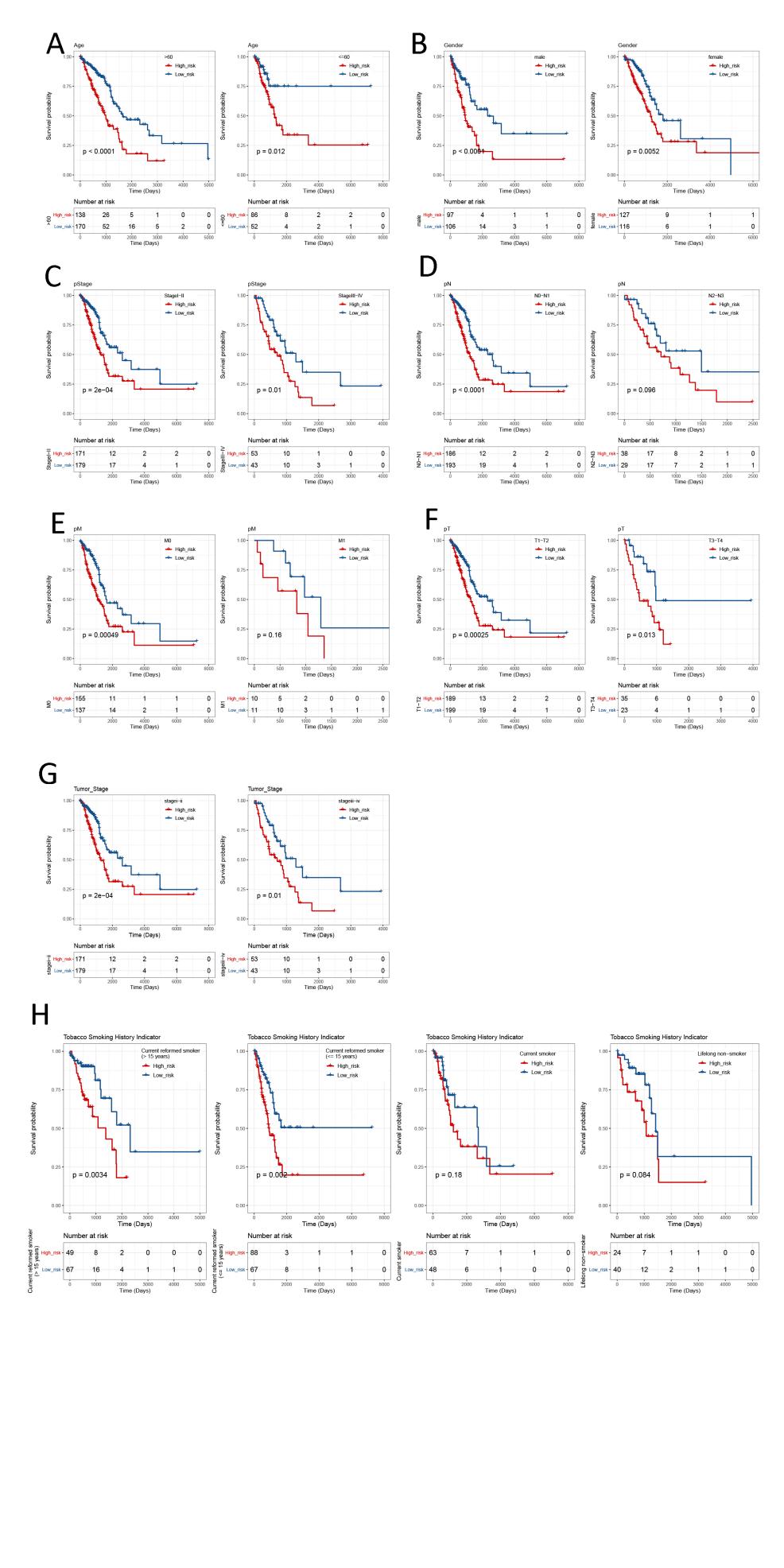


The relationship between clinicopathologic features and survival was analyzed by stratified survival analysis of high and low risk clinical factors. The results showed that there were significant differences in all clinical traits except for pN2-pN3, M1, current smoker and Lifelong non-smoker in high-and low-risk groups.
